# The Effect of Physical Exercise during COVID-19 Lockdown

**DOI:** 10.3390/healthcare11111618

**Published:** 2023-06-01

**Authors:** Pasquale Ricci, Margherita Pallocci, Michele Treglia, Serafino Ricci, Rosaria Ferrara, Claudia Zanovello, Pierluigi Passalacqua, Felice Marco Damato

**Affiliations:** 1 Department of Human Anatomy, Histology, Forensic Medicine and Orthopedics (S.A.I.M.L.A.L.), Legal Medicine Section, “Sapienza” University of Rome, 00185 Rome, Italy; 2 Department of Biomedicine and Prevention, University of Rome “Tor Vergata”, 00133 Rome, Italy; 3 Department of Public Health and Infectious Diseases, “Sapienza” University of Rome, 00185 Rome, Italy

**Keywords:** COVID-19, pandemic, mental health, infectious diseases, physical exercise

## Abstract

The COVID-19 pandemic has exerted an effect on the general population that went over every expectation. To explore the effect of physical exercise (PE) during the national lockdown in Italy, a survey was drawn up and administered to a sample composed of 208 subjects. The questionnaire comprised 81 multiple-choice items, including sociodemographic data, health-related questions, and assessment of physical exercise, satisfaction with life, depression, and personality. The purpose of this study is to explore the role of physical exercise during the outbreak following the present hypothesis: first, if a link exists between the time spent on physical exercise during the lockdown and the perceived health condition, depressive and somatic symptomatology, and life satisfaction; second, to find associations among the SF-12 component summaries and the other psychological outcomes; and finally, to explore how physical and psychological variables are predictive of PCS-12 and MCS-12. The results showed that both vigorous and moderate physical exercise was strongly correlated with psychological variables, with statistically significant negative correlations found between age and physical exercise. Additionally, significant positive correlations were observed between physical exercise and mental health indices, such as MCS-12 and SWLS, whereas negative correlations were found with BDI, PCS-12, and SOM-H. The correlation analysis also revealed that physical and individual mental health summaries were associated with psychological outcomes, with statistically significant negative correlations found between PCS-12 and MCS, PCS-12 and SOM-H, and MCS-12 and BDI scores. Regression analysis showed that physical activities and psychological status both had a direct influence on perceived mental and physical well-being during the lockdown, accounting for 56.7% and 35.5% of the variance, respectively. The *p*-values for the significant correlations ranged from <0.05 to <0.01. Overall, these findings highlight the importance of physical exercise and psychological well-being in maintaining good health during the pandemic.

## 1. Introduction

COVID-19 (SARS-CoV-2) is a coronavirus disease that spread rapidly among people, resulting in a large epidemic that has caused a large number of deaths. Epidemiologic data show how, since the first detection of COVID-19 infection in the Chinese city of Wuhan, the spreading of the disease involved many countries, which adopted measures of lockdown such as the ones adopted by the Chinese on 21 January 2019 in many cities of Hubei region [1]. As regards to Italy, the first confirmed case of COVID-19 infection was observed on 31 January 2020 in Lombardy, which became the centre of the Italian pandemic. The travel of citizens affected by COVID-19 across the country led to a rapid increase in cases, forcing the Italian government to adopt the national lockdown measures to prevent the diffusion of the virus from 11 March to 3 May 2020 [2,3]. Indeed, Italy scored the highest case fatality rate (CFR), with 14.5% of the confirmed cases passing away, compared with 1.8% of cases in South Korea and 4% of cases in China (data refers to 4 April 2020) [4]. The national quarantine state promulgated on 11 March represents the largest suspension of constitutional rights in Italian republican history, with the adoption of many restrictive measures. For example, the government banned all unnecessary intra-national travels and disposed of limitations on citizens’ free movements (e.g., getting out of the house for pet needs and outdoor individual fitness activities were admitted), non-essential commercial and retail markets, unnecessary industry activities, and closure of public events. The lockdown exerted enormous psychological effects on the population that had to deal with a drastic change in their daily habits, with potential long-term consequences [5]. Reviews of the literature have identified the psychological symptomatology among quarantined individuals, with a large prevalence of post-traumatic and depressive symptoms, anxiety, feelings of anger, and stress [6,7,8,9]. Currently, a survey conducted on the Italian population during the national lockdown in Italy [10] shows that the female gender, negative relationships, separations, and a history of stressful situations are associated with anxiety, depression, and stress. Furthermore, the aforementioned study found another link between a history of medical issues and an increase in anxiety and depression. According to the international literature, physical exercise brings a significant enhancement to health and the function of both muscles and bones, increasing strength and endurance and reducing the risk of chronic diseases (such as obesity and diabetes). Additionally, it improves the psychological well-being, raising the threshold tolerance for stress, anxiety, and depression [11,12]. On the same issue, Da Silveira et al. [13] have traced a theoretical basis for the study of the immune system response, focusing on the benefits provided by exercise during the COVID-19 pandemic; moreover, the authors have called on the scientific community to deepen studies about lifestyles among the population to promote health and well-being. Additionally, physical exercise has been shown to have a positive impact on mental health, with various populations benefiting from it, including healthy individuals and those with psychological conditions. Caponnetto et al. [14] conducted a review investigating the effects of physical exercise on mental health, from cognitive improvements to addiction risk, as well as the impact of the COVID-19 pandemic on exercise addiction and associated disorders. The review found that physical exercise can enhance cognitive functions in healthy individuals, children with ADHD, and patients with a schizophrenic spectrum disorder or bipolar disorder. However, exercise addiction can be linked to low levels of education, low self-esteem, eating disorders, and body dysmorphism. Additionally, people with lower traits and intolerance of uncertainty exhibit a strong association between COVID-19 anxiety and compulsive exercise and an eating disorder. These findings emphasize the importance of adequately assessing psychological distress and personality characteristics linked to exercise addiction. Moreover, Fallon et al. [15] found that individuals with chronic pain reported higher pain severity and reduced levels of physical exercise during COVID-19 lockdowns, leading to increased anxiety and a depressed mood. Pain catastrophizing was identified as a key factor mediating the relationship between decreased mood and pain severity during the lockdown. Remote pain management programs targeting the reduction of pain catastrophizing and promoting health behaviours, such as physical exercise, could benefit individuals with chronic pain. Furthermore, Zhang and Velez [16] revealed that inactive individuals were more likely to experience psychological distress, depression, and anxiety than highly active individuals during the pandemic. COVID-19 had a greater impact on reducing the chances of less active individuals engaging in physical exercise outside and in public spaces. Highly active individuals’ exercise locations changed less and their exercise frequency at home increased. Interventions aimed at enhancing physical exercise among less active individuals may help address the mental health crisis caused by the pandemic. On the other hand, Peng et al. [17] added a new perspective by introducing virtual reality fitness, which they found to have a positive effect on exercise, general well-being, and physical-psychological health, providing a promising solution to promote exercise and general well-being during periods of exercise restrictions. Although the effects of physical exercise are generally studied among elder subjects affected by neurodegenerative diseases, studies have shown that these benefits are much the same between frail and non-frail people [18]. The purpose of this study is to explore the role of physical exercise during the pandemic through the investigation of different issues: firstly, the existence of a link between the time spent in physical activity during the lockdown and the perceived health condition, depressive and somatic symptomatology, and life satisfaction; secondly, we expect to find associations among the SF-12 component summaries and the other psychological outcomes; finally, the last purpose is to explore how both physical and psychological variables are predictive of PCS-12 and MCS-12.

## 2. Materials and Methods

### 2.1. Procedures

Data were collected via an online survey from 22 April to 15 May 2020, administered during the COVID-19 national lockdown in Italy. We evaluated the psychological factors and physical exercise habits of the subject; the anonymous questionnaire in the Italian language was assembled by the research team, inspired by articles published by Zhang et al. [19] and Chang [20]. The management of the online surveys used the Google Forms platform, accessible by the participants using a specific link sent on WhatsApp, Telegram, or by e-mail.

### 2.2. Sample

The final sample consisted of 208 subjects, obtained from an initial sample of 229, through the application of the following inclusion criteria: (a) being at least 18 years old, (b) completing the questionnaire without omitting items, especially if belonging to psychological measures or items related to exercise, and (c) subscribing to the informed consent submitted at the beginning of the questionnaire. One subject was not admitted to the final sample because he/she was younger than 18 years old, and 20 subjects did not subscribe to the informed consent. The informed consent explained the procedures of the survey: participants could quit or interrupt the survey at any moment, without any specific reason. Participation was entirely voluntary and participants could withdraw at any time without penalty. The privacy of participants was guaranteed by anonymity, and sensitive data was stored securely on Google Forms, with limited access granted only to the project manager through a password-protected account. The data were handled in accordance with privacy laws, including the Italian Data Protection Code (Legislative Decree n. 196/2003) and the European General Data Protection Regulation (GDPR). The sampling method used was convenience sampling, where participants were recruited based on their availability and accessibility to participate in the study. The informed consent explained that participants could stop completing the questionnaire at any time without providing a reason. However, some participants were excluded based on certain criteria, such as expressing disagreement with the informed consent and omitting answers belonging to a specific psychological measure or items related to vigorous and moderate physical exercise.

### 2.3. Questionnaire Development

The online questionnaire consisted of 81 multiple-choice items, of which 16 were dedicated to sociodemographic data such age, gender, education, occupation, place of birth, sedentary behaviour, presence of pathologies, acquaintance/personal contact with COVID-19, possession of pets, and cohabitation with family members or flatmates. The remaining items included 12 questions from the short-form health survey (SF-12) [21,22,23,24], 11 questions about daily activities and physical exercise, 5 questions from the satisfaction with life scale (SWLS) [25], the complete administration of the 21 questions from the Beck depression inventory (BDI) [26,27,28], and 16 items selected from the personality assessment inventory (PAI) [29].

#### 2.3.1. Physical Exercise

To evaluate the weekly physical exercise in the survey two questions about how many days were devoted to vigorous and moderate physical activity were included. Moderate physical exercise (MPE) refers to all sports activities requiring moderate physical exertion carried out in leisure time, such as cycling, swimming at a low rate, or playing doubles tennis. Subjects have been asked to think about MPE carried out for at least 10 consecutive minutes. Similarly, vigorous physical exercise (VPE) refers to sports activities such as aerobics, running, cycling fast, or swimming at a high rate. In addition, the questionnaire (see Supplementary Materials) analysed the days dedicated to activities such as walking, movements for daily activities, and the use of vehicles. Questions required respondents to count how many days, from 0 to 7, were usually dedicated to the exercise indicated by the item. Cronbach’s alpha value for the physical exercise items is 0.74.

#### 2.3.2. Individual Health Status

The Italian adaption of the SF-12 questionnaire [24] was used to examine individual health. SF-12 is a self-report questionnaire and includes 12 multiple-choice items assessing 8 different dimensions grouped in two composite scores of physical and mental scales (PCS and MCS), with a score ranging from 0 to 100 [22]. An excel sheet was completed to calculate both the MCS and PCS according to the guidelines of the Italian Technical Manual cited before. SF-12 is a factorial reduction-derived questionnaire, and each item loads in a different mode on both physical and mental composite scores. Each answer was recodified with specific weighted scores for MCS and PCS.

#### 2.3.3. Depressive Symptomatology

The 21-item BDI-II [27] measures the severity of self-reported depression in adolescents and adults. Each item is rated on a 4-point scale from 0 to 3, and the full scale ranges from 0 to 63. The Italian version of BDI-II is comparable with the original edition, with a Cronbach’s Alphas of 0.86 for the mental factor and 0.65 for the secondary factors [28]. For the current study, the Cronbach’s alpha value used for full item composition is 0.88. For the current study, the prevalence of depressive pathology among participants was not considered.

#### 2.3.4. Somatic Symptomatology

To assess somatic symptomatology, 16 items from the SOM scale of the PAI were selected. These items are referred to on the two subscales SOM-S and SOM-H; both are composed of 8 items. SOM-S focuses on common physical complaints and somatizations; SOM-H evaluates the self-perception of one’s health status, health problems, and efforts to improve health. Each item consists of a 4-point Likert scale ranging from 0 (not at all true) to 3 (very true), and the total raw scores for subscales can range from 0 to 24 [29,30]. An excel worksheet was compiled using item characteristics published in the Italian version of the technical manual [31] to calculate the raw scores of the SOM-S and SOM-H. Furthermore, the PAI median Cronbach’s alpha for subscales reported by Pignolo et al. [32] is 0.78. For the present study, the alpha values are 0.80 for SOM-H and 0.52 for SOM-S.

#### 2.3.5. Life Satisfaction

Life satisfaction was assessed through the administration of the self-reported questionnaire SWLS (satisfaction with life scale) [25]. The SWLS asks the subject to express the level of agreement with five statements rated from 1 (strongly disagree) to 7 (strongly agree). The score can range from 5 up to 35 and provides information about global life satisfaction. Higher scores represent better life satisfaction. Cronbach’s alpha for the Italian version of the SWLS [33] is 0.85 while for the present study it is 0.56.

### 2.4. Statistical Analysis

To verify the study purposes, statistical analyses were performed using SPSS 25. For both the first and the second aim of the current study, multiple Spearman’s rho correlations were performed. To analyse the effect of the variables on perceived well-being, two hierarchical regression analyses were carried out. In the first hierarchical regression, with MCS as the dependent variable, a block of three predictors (walking activity, vehicle usage, and necessary activities) were entered in the first step, followed by age (step two), vigorous and moderate physical activities (step three), BDI-II, SOM-H, SOM-S (step four), PCS-12 (step five), and SWLS score in the sixth and final step. The same procedure was used for the second hierarchical regression, with PCS-12 as the dependent variable and MCS-12 inserted in the fourth step.

## 3. Results

### 3.1. Descriptive Statistics

Table 1 reports data on the 208 participants, consisting of 144 females (69.2%) and 64 males (30.8%) with an age ranging between 19–75 years (M = 34.61, SD = 14.1). The majority of participants had attained a high school diploma (42.3%) or a graduate degree (53.8%), whereas only a small number reported completing middle school (2.4%) or other types of education (1.4%). In terms of place of birth, most participants were from the south and islands of Italy (50.5%), followed by the central region (32.2 %), Northern Italy (15.4%), and foreign countries (1.9%). The most common occupation was employee (38.9%), followed by students (26.9%) and freelancers (17.8%), whereas some participants reported being unemployed or retired (16.3%). The participants reported engaging in moderate physical exercise (MPE) more frequently (M = 2.22, SD = 2.2) than walking (M = 2.04, SD = 2.1) and vigorous physical exercise (VPE) (M = 1.89, SD = 2.17). The mean score for the Beck depression inventory (BDI) was 10.58 (SD = 7.9), indicating low levels of depression, and the mean score for the physical component summary (MCS-12) was 37.46 (SD = 10.8), suggesting lower levels of mental well-being. The participants reported a moderate level of somatic symptoms (SOM-S, M = 6, SD = 4.1) and a relatively low level of somatic health (SOM-H, M = 4.65, SD = 4.3). Finally, the participants reported a relatively high level of life satisfaction, as indicated by the mean score of 21.32 (SD = 4.9) on the satisfaction with life scale (SWLS).

The association between days devoted to physical exercise, health, and psychological outcomes.

According to the first aim, both vigorous and moderate physical activity reached strong correlations with the psychological variable. The results of all correlations can be found in Appendix A (Table A1 and Table A2) and Appendix B (Table A3).

#### 3.1.1. Vigorous Physical Activity vs. Psychophysiological Variables

The results suggest a significant negative correlation between age and VPE (r = −0.228, *p* < 0.01), indicating that older individuals engage in less vigorous physical activity. Furthermore, a significant negative correlation was found between BDI and VPE (r = −0.184, *p* < 0.05). On the other hand, a significant positive correlation was observed between VPE and MCS-12 (r = 0.226, *p* < 0.01). Similarly, VPE was positively correlated with SWLS (r = 0.218, *p* < 0.01). Additionally, there was a significant negative correlation between VPE and BDI (r = −0.130, *p* < 0.05). However, no significant correlations were found between physical exercise and PCS-12, SOM-S, and SOM-H.

#### 3.1.2. Moderate Physical Activity vs. Psychophysiological Variables

For moderate physical exercise (MPE), the correlation matrix reveals that there was a statistically significant negative correlation between age and MPE (r = −0.162, *p* < 0.05). Additionally, there was a statistically significant negative correlation between BDI and MPE (r = −0.138, *p* < 0.05). However, no significant correlations were found between MPE and PCS-12, SOM-S, and SOM-H. MPE was positively correlated with MCS-12 (r = 0.165, *p* < 0.05). Finally, MPE was positively correlated with SWLS (r = 0.153, *p* < 0.05).

#### 3.1.3. Vigorous and Moderate Physical Activity vs. Age

The results indicate that there was a stronger negative correlation between age and VPE (r = −0.228, *p* < 0.01) compared with the weaker negative correlation between age and MPE (r = −0.162, *p* < 0.05), suggesting that the number of days dedicated to moderate and/or vigorous physical activity has no relationship with age.

### 3.2. Association between the MCS-12 and PCS-12 Indices with Psychological Variables

Correlation analysis shows the existence of an association between physical and individual mental health summaries and the psychological outcomes for somatic symptomatology, depressive symptoms, and life satisfaction.

#### 3.2.1. PCS-12 vs. Psychological Variables

For MCS-12, there was a statistically significant negative correlation between MCS-12 and BDI score (r = −0.165, *p* < 0.05). Additionally, there was a statistically significant negative correlation between MCS-12 and SOM-S score (r = −0.260, *p* < 0.01). Finally, there was a statistically significant positive correlation between MCS-12 and SWLS score (r = 0.265, *p* < 0.01). No significant correlation was found between PCS-12 and BDI or SOM-S.

#### 3.2.2. MCS-12 vs. Psychological Variables

For MCS-12, there was a statistically significant negative correlation between MCS-12 and BDI score (r = −0.165, *p* < 0.05). Additionally, there was a statistically significant negative correlation between MCS-12 and SOM-S score (r = −0.260, *p* < 0.01). Finally, there was a statistically significant positive correlation between MCS-12 and SWLS score (r = 0.265, *p* < 0.01).

#### 3.2.3. PCS-12 and MCS-12 vs. Age

The correlations show a statistically significant negative correlation between age and PCS-12 (r = −0.270, *p* < 0.01). On the other hand, there was a statistically significant positive correlation between MCS-12 and PCS-12 (r = 0.312, *p* < 0.01). No significant correlations were found between age and MCS-12.

### 3.3. Effect of Physical and Psychological Outcomes on Individual Health Status

**Figure 1:**
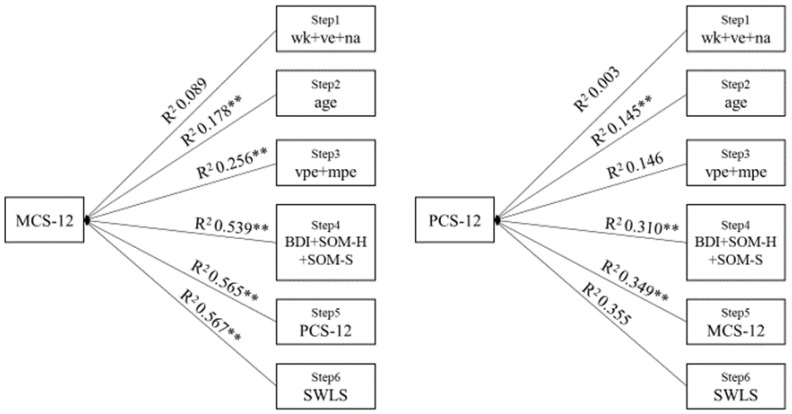
Hierarchical regression analyses showing the accounted for amount of variance by necessary activity during the lockdown, physical exercise, and psychological outcome. WK = Walking Activity; VE = Vehicle Usage; NA = Necessary Act.; VPE = Vigorous Phys. Exercise; MPE = Moderate Phys. Exercise. ** Significant modification of R^2^, *p* < 0.01.

As shown in Figure 1, for mental components, the days devoted to the walking activity, vehicle usage, and necessary activity account for 8.9% of the variance in step one. In the second and third steps, whereas age accounts for a significant 8.9%, vigorous and moderate physical activity add 7.9% of the additional variance. In the fourth step, the entering of the psychological outcomes produced an increase of 28.3% of the explained variance. Physical components entered in the fifth step added another 2.6% of the variance. In the final step, the SWLS accounted for a non-significant 0.2%. The full regression model accounted for 56.7% of the variance in perceived mental well-being.

For physical health status, the first set of variables accounts for 0.3% of the variance in step one. Age produced a significant increase of 14.2% of the variance in step two. In the third step, physical activities add a non-significant 0.01% to the accounted variance. In the fourth step, depressive and somatic outcomes account for a significant 16.4% of the additional variance. In the final steps, mental components and the SWLS add a significant 3.9% and a non-significant increase of 0.06% on the accounted variance, respectively. The full model accounts for 35.5% of the variance in perceived physical well-being. Most of all, these results suggest that psychological status and physical activities both had a direct influence on perceived mental and physical well-being during the lockdown. The entire analysis can be found in Appendix B.

## 4. Discussion

Considering what was shown by the correlation analysis, participants who devoted a higher number of days to VPE and MPE during lockdown had a better mental health status; they tend to represent themselves as less depressed and more satisfied than people with a lower frequency of physical exercise. Staying on this topic, the findings of the present study are in line with multiple studies that have pointed out how indoor physical exercise has presented a massive protective factor for health during the national lockdown [34,35], even for older subjects [36,37] and other fragile categories [38,39,40,41]. It is also reasonable to consider that some exercise habits, such as outdoor exercise, can be less healthy considering the role of environmental pollutants after the end of the lockdown measures and its effect observed on indoor and outdoor workers [42,43]. The non-association between days spent engaging in physical exercise probably means that the somatic symptomatology, also considering that the PAI’s items related to clinical assessment, is independent of physical exercise. Concerning the second goal, the investigation has shown that a subject with a higher score for physical index tended to display lower focus on their health and fewer physical complaints and somatizations. On the other hand, participants who obtained a higher score for the mental index tended to describe themselves as more satisfied and showed lower depressive and somatic symptoms. These data point to the same problems that were also found among very young populations that particularly suffered from social distancing measures [44] that naturally changed the normal lifestyle of the whole population. A previous study, conducted by Skurvydas et al. [45], investigated the relationship between physical activity, health, and quality of life in older adults. The study found that physical activity was positively correlated with quality of life and health-related variables such as body mass index and blood pressure. Additionally, the study found that the psychological variables of depression and anxiety were negatively correlated with physical activity. The results of the present study are in line with Skurvydas et al.’s findings, as both studies found that physical exercise and psychological variables were significantly associated with individual health status. However, the present study’s focus on the COVID-19 lockdown highlights the importance of physical exercise and mental health during times of crisis and social isolation. Future research should continue to investigate the associations between physical exercise, psychological variables, and individual health status, particularly during times of societal stress and change. The impact of isolation tends to change depending on the age of the subjects; for example, elderly participants happened to more frequently show a worse physical state than a better mental health state; in contrast, younger subjects had inverse mental and physical characteristics compared with older subjects. Moreover, in terms of linear correlation, a subject with a higher PCS tended to have a low MCS and vice versa. Finally, hierarchical regressions performed with both PCS-12 and MCS-12 as dependent variables showed that the awareness of depressive symptoms and somatic problems are the two main predictors for the whole health status. This statement is confirmed by analysing the hierarchical regression, in which the combination of the three variables produces a significant increase in the explained variance. In particular, the somatic field appeared to be the most relevant factor for people during the lockdown; thoughts related to the somatic areas have probably exerted some sort of influence, diverting the psychological resources to a set of concerns and thoughts related to the possible negative consequences of forced confinement. Comparing the results with those of the Brazilian population [46], we find that the data obtained are in line with the data analysed in this study; on the other hand, our results are different from a Chinese study, which showed that people who exercised more hours a day were less satisfied than people with lower exercise frequencies [19]. In comparison with Gallè et al. [47], who investigated the impact of the COVID-19 pandemic on the physical and mental health of the general population, our study specifically focused on the relationship between physical exercise, psychological outcomes, and individual health status during the lockdown. Our findings are consistent with Gallè et al., who found a strong relationship between physical exercise and mental health, as well as the impact of psychological factors on perceived health outcomes. However, our study adds to the literature by providing more specific insights into the relationship between different types of physical exercise and psychological variables. Additionally, our study suggests that both physical and psychological outcomes are important factors that influenced the perceived health status during the lockdown, which highlights the need for interventions that address both domains to promote overall well-being. This study has some limitations. First, the number of participants is not a representative sample of the Italian population, so the results of this study do not allow statistical inference to the general population. Secondly, the high scores obtained for the BDI-II may be the result of false positives because some items in the inventory investigate the naturally modified aspects of the restrictive measures adopted during the pandemic. Another limitation is the analysis of sedentary behaviour, which appears to have increased in the Italian population during quarantine [47] and could provide more potentially useful information to deepen the analysis carried out using the results obtained in the present study. The questions investigated how many times the respondent spent in sedentary activity but were not focused on the precise amount of time in terms of minutes or hours.

## 5. Conclusions

The results of the present study show how physical exercise (both moderate and vigorous) is closely associated with a good self-perception of mental well-being. These results lead to the consideration that, in general, physical exercise has been a good protective factor during the lockdown. It was found that, at the level of hierarchical regression, the effect exerted by more recreational activities, such as simple walking combined with exercise, produces an effective benefit for perceived mental health. In contrast, variables related to the measure of symptoms of depression and psychosomatic disorders significantly affected subjects’ physical self-perception. Future studies should be used to establish the ideal amounts of exercise, sedentary activities, and dietary habits to ensure the best possible health for subjects restricted from freedom due to infectious diseases.

## Figures and Tables

**Table 1:** Descriptive statistics.

	Group	M (SD)	n (%)
Gender	Female		144 (69.2%)
	Male		64 (30.8%)
Age	Min–Max (19–75)	34.61 (14.1)	208 (100%)
Education	Middle school		5 (2.4%)
	high school		88 (42.3%)
	Graduate		112 (53.9%)
	Other (Primary school, profession certification)		3 (1.4%)
Place of birth	South and islands (Italy)		105 (50.5%)
	Centre (Italy)		67 (32.2%)
	North (Italy)		32 (15.4%)
	Foreign		4 (1.9%)
Occupation	Employee		81 (39.0%)
	Freelancer		37 (17.8%)
	Student		56 (26.9%)
	Other (Unemployed, retired)		34 (16.3%)
Movements for necessary activities	Min–Max (0–7)	2.54 (2.2)	
Walking activity	Min–Max (0–7)	2.04 (2.1)	
Vehicle usage	Min–Max (0–7)	0.91 (1.6)	
Vigorous physical exercise (VPE)	Min–Max (0–7)	1.89 (2.17)	
Moderate physical exercise (MPE)	Min–Max (0–7)	2.22 (2.2)	
BDI	Min–Max (0–37)	10.58 (7.9)	
PCS-12	Min–Max (29.08–66.12)	54.84 (6.07)	
MCS-12	Min–Max (11.30–58.83)	37.46 (10.8)	
SOM-H (Raw scores)	Min–Max (0–20)	4.65 (4.3)	
SOM-S (Raw scores)	Min–Max (0–19)	6 (4.1)	
SWLS	Min–Max (9–33)	21.32 (4.9)	

## Data Availability

The data presented in this study are available on request from the corresponding author.

## Supplementary Materials

The following supporting information can be downloaded at: https://www.mdpi.com/article/10.3390/healthcare11111618/s1. Questionnaire.

## Appendix A

**Table A1:** Spearman rho correlation between days spent engaging in physical exercise, age, and phycological indexes. Significance ** *p* < 0.01; * *p* < 0.05.

	AGE	BDI	PCS-12	MCS-12	SOM-H	SOM-S	SWLS
VPE	−0.228 **	−0.184 *	0.033	0.226 **	−0.023	−0.130	0.218 **
	0.001	0.008	0.640	0.001	0.755	0.061	0.002
MPE	−0.162 *	−0.138 *	0.043	0.165 *	−0.015	−0.127	0.153 *
	0.020	0.046	0.533	0.017	0.830	0.069	0.028

**Table A2:** Spearman rho correlations between SF-12 scales, age, and psychological indexes. Significance ** *p* < 0.01; * *p* < 0.05.

	AGE	BDI	PCS	MCS	SOM-H	SOM-S	SWLS
PCS-12	−0.270 **	−0.027	1	−0.230 **	−0.263 **	−0.355 **	0.074
	0.000	0.694		0.001	0.000	0.000	0.289
MCS-12	0.312 **	−0.165 *	−0.230 **	1	−0.072	−0.260 **	0.265 **
	0.000	0.017	0.001		0.300	0.000	0.000

## Appendix B

**Table A3:** Hierarchical regression analyses showing the accounted for amount of variance by necessary activity during the lockdown, physical exercise, and psychological outcome.

Physical and Psychological Outcome Measure	R	R^2^	df	F
MCS-12				
WK + VE + NA	0.298	0.089	204	6.630
age	0.421	0.178	202	21.888 **
VPW + MPE	0.506	0.256	201	10.610 **
BDI + SOM-H + SOM-S	0.734	0.539	198	40.487 **
PCS-12	0.752	0.565	197	11.768 **
SWLS	0.753	0.567	196	0.865
PCS-12				
WK + VE + NA	0.053	0.003	204	0.189
age	0.065	0.145	202	33.808 **
VPE + MPE	0.383	0.146	201	0.140
BDI + SOM-H + SOM-S	0.557	0.310	198	15.709 **
MCS-12	0.591	0.349	197	11.768 **
SWLS	0.596	0.355	196	1.798

WK = Walking Activity; VE = Vehicle Usage; NA = Necessary Act.; VPE = Vigorous Phys. Exercise; MPE = Moderate Phys. Exercise. ** Significant modification of R^2^, *p* < 0.01
