# Supplementary material for: The Effect of Physical Exercise during COVID-19 Lockdown
Source: Healthcare (Basel). 2023 Jun 1;11(11):1618. doi: 10.3390/healthcare11111618 (PMC10252320; doi:10.3390/healthcare11111618)
Supplement: Supplementary file 1 [file healthcare-11-01618-s001.zip › healthcare-2329782-supplementary.pdf]

questionnaire

GENERAL DATA

1. Place of birth (home country or region of Italy)

   Contrassegna solo un ovale.

   - Northern region of Italy
   - Central Italy region
   - Southern region and islands of Italy
   - Foreign Country

2. OCCUPATION

   Contrassegna solo un ovale.

   - Employee
   - Freelancer
   - Student
   - Altro: __________

3. AGE

   ____

4. Gender *

Contrassegna solo un ovale.

- Male
- Female
- I prefer not to specify it
- Altro: ____________________

5. EDUCATION

Contrassegna solo un ovale.

- Primary School
- Middle School
- High School
- Professional High School
- Graduated
- Post Graduated

Section A

INSTRUCTIONS: This section is intended to assess what you think about your health. The information
collected will allow you to be kept up to date on how you feel and how you are managing to perform your
usual activities. Answer each question on the questionnaire indicating your answer as shown from time to time. If you do not feel certain of the answer, make the choice that still seems best to you. (SF-12)

6. In general, would you say your health is:

Contrassegna solo un ovale.

- Excellent
- Very Good
- Good
- Fair
- Poor

7. Does your health currently limit you in performing moderate strenuous activities (such as moving a table, Pushing a vacuum cleaner, bowling, or playing golf) ?

Contrassegna solo un ovale.

- Yes, Limited a Lot
- Yes, Limited a Little
- No, Not limited at All

8. Does your health currently limit you in Climbing several flights of stairs ?

Contrassegna solo un ovale.

- Yes, Limited a Lot
- Yes, Limited a Little
- No, Not limited at All

9. In the past 4 weeks, have you Accomplished less than you would have liked at work or in other daily activities as a result of your physical health?

Contrassegna solo un ovale.

- Yes
- No

10. In the past 4 weeks, have you limited in the kind of work or other activities as a result of your physical health?

Contrassegna solo un ovale.

- Yes
- No

11. In the past 4 weeks, have you had Accomplished less than you would like at work or in other daily activities because of your emotional state (such as feeling depressed or anxious)?

Contrassegna solo un ovale.

- Yes
- No

12. In the past 4 weeks, have you not done your work or other activities with your usual attention?

Contrassegna solo un ovale.

- Yes
- No

13. During the past 4 weeks, how much did pain interfere with your normal work (including both work outside the home
and housework)?

Contrassegna solo un ovale.

- Not at all
- A little Bit
- Moderately
- Quite a Bit
- Extremely

14. Have you felt calm and peaceful?

Contrassegna solo un ovale.

- All of the Time
- Most of the time
- A Good Bit of the Time
- Some of the Time
- A little of the Time
- None of the Time

15. Did you have a lot of energy?

Contrassegna solo un ovale.

- All of the Time
- Most of the time
- A Good Bit of the Time
- Some of the Time
- A little of the Time
- None of the Time

16. Have you felt downhearted and blue?

Contrassegna solo un ovale.

- All of the Time
- Most of the time
- A Good Bit of the Time
- Some of the Time
- A little of the Time
- None of the Time

17. Have you felt downhearted and blue?

Contrassegna solo un ovale.

- All of the Time
- Most of the time
- Some of the Time
- A little of the Time
- None of the Time

Section B

We are interested in learning about the types of physical activities that people do as part of their daily. The questions will cover the time you spent on physical activities in the last seven days. Kindly answer each question even if you do not consider yourself an active person.

18. During the past seven days, on how many days did you walk for necessary activities? (0 to 7)

Contrassegna solo un ovale.

- 0 ◯
- 1 ◯
- 2 ◯
- 3 ◯
- 4 ◯
- 5 ◯
- 6 ◯
- 7 ◯

19. During the past seven days, on how many days did you ride in a motor vehicle such as train, bus, car, or streetcar? (0 to 7)

Contrassegna solo un ovale.

- 0
- 1
- 2
- 3
- 4
- 5
- 6
- 7

20. During the past seven days, on how many days did you walk from one place to another? (0 to 7)

Contrassegna solo un ovale.

0

1

2

3

4

5

6

7

21. During the past seven days, on how many days did you engage in moderate physical activity in your free time? (0 to 7)

all sports activities that require moderate physical exertion that you have done in your spare time, such as biking or swimming at a leisurely pace, or playing doubles tennis. Think again only of those moderate physical activities that you have done for at least 10 consecutive minutes.

Contrassegna solo un ovale.

0

1

2

3

4

5

6

7

22. During the past seven days, on how many days did you engage in vigorous physical activity in your free time?(0 to 7)

First, think of vigorous sports activities you have done, such as aerobics, running, bicycling fast, or swimming fast.

Contrassegna solo un ovale.

0
1
2
3
4
5
6
7

23. In most ways my life is close to my ideal

Contrassegna solo un ovale.

	Strongly disagree
1	◯
2	◯
3	◯
4	◯
5	◯
6	◯
7	◯
	Strongly agree

24. The conditions of my life are excellent

Contrassegna solo un ovale.

	Strongly disagree
1	◯
2	◯
3	◯
4	◯
5	◯
6	◯
7	◯
	Strongly agree

25. I am satisfied with my life

Contrassegna solo un ovale.

Strongly disagree
1 ◯
2 ◯
3 ◯
4 ◯
5 ◯
6 ◯
7 ◯
Strongly agree

26. So far I have gotten the important things I want in life

Contrassegna solo un ovale.

Strongly disagree	
1	⬭
2	⬭
3	⬭
4	⬭
5	⬭
6	⬭
7	⬭

Strongly agree

27. If I could live my life over, I would change almost nothing

Contrassegna solo un ovale.

	Strongly disagree
1	◯
2	◯
3	◯
4	◯
5	◯
6	◯
7	◯
	Strongly agree

Some item sections were omitted to avoid copyright infringement. Specifically, the items were from the BDI-II instruments and the SOM sub-scales of the PAI questionnaire.

Questi contenuti non sono creati né avallati da Google.

Google Moduli
